# The whole mitochondrial genome of the mangrove crab, *Metopograpsus frontalis* (Miers, 1880) (Decapoda, Grapsidae) and its phylogenetic relationship

**DOI:** 10.1080/23802359.2018.1450685

**Published:** 2018-03-14

**Authors:** Mengyun Guan, Xinming Liu, Fan Lin, Zhuofang Xie, Hanafiah Fazhan, Mhd Ikhwanuddin, Huaqiang Tan, Hongyu Ma

**Affiliations:** a Guangdong Provincial Key Laboratory of Marine Biotechnology, Shantou University, Shantou, China;; b Marine Resource and Environment Research Institute, Guangxi Academy of Oceanography, Nanning, China;; c Institute of Tropical Aquaculture, Universiti Malaysia Terengganu, Kuala Terengganu, Malaysia

**Keywords:** *Metopograpsus frontalis*, mitochondrial genome, phylogeny

## Abstract

In this study, we sequenced and analyzed the whole mitochondrial genome of *Metopograpsus frontalis* Miers, 1880 (Decapoda, Grapsidae). The circular genome is 15,587 bp in length, consisting of 13 protein-coding genes, 22 transfer RNA genes, 2 ribosomal RNA genes, as well as a control region. Both *atp8*/*atp6* and *nad4L*/*nad4* share 7 nucleotides in their adjacent overlapping region, which is identical to those observed in other Grapsidae crabs. The genome composition and gene order follow a classic crab-type arrangement regulation. The phylogenetic analysis suggested that Grapsidae crabs formed a solid monophyletic group. The newly described mitochondrial genome may provide genetic marker for studies on phylogeny of the grapsid crabs.

The mangrove crab, *Metopograpsus frontalis* lives in mangroves and intertidal areas of eastern Indian and western Pacific Oceans, from Singapore to southern China (Fratini et al. 2018). This species is an opportunistic feeder with good predatory ability, potentially important to estuarine food webs (Poon et al. 2010). The taxonomy of the genus has been questioned due to minor diagnostic morphological differences among species (Fratini et al. 2018). The whole mitochondrial genome sequence can facilitate the validation of taxonomic classification (Ma et al. 2015). The present study reports the determination of the whole mitochondrial genome of *M. frontalis* and analyzes its phylogenetic relationship. This is the third Grapsidae crab whose whole mitogenome is sequenced to date.

Specimens of *M. frontalis* were collected from Weizhou Island (21.0234°N, 109.0940°E), Guangxi province, China, and they were deposited at the Marine Biology Institute, Shantou University, Shantou, China. Total genomic DNA was isolated from the muscle tissue. Long and conventional PCRs were employed to obtain the whole mitochondrial genome sequence. The mitogenome was assembled, annotated and finally submitted into GenBank database under the accession number of MH028874.

The whole mitogenome sequence of *M. Frontalis* is 15,587 bp in length, containing 13 protein-coding genes, 22 transfer RNA genes, 2 ribosomal RNA genes and 1 putative control region. Of the 37 genes, twenty-three are encoded by the plus strand and the remainders by the minus strand. Both *atp8*/*atp6* and *nad4L*/*nad4* share 7 nucleotides in their adjacent overlapping region, which is identical to those observed in other Grapsidae crabs, such as *Pachygrapsus crassipes* (Yu et al. 2014), but different with the other crabs, such as *Scylla paramamosain* (Ma et al. 2013) and *Charybdis feriata* (Ma et al. 2015). For the 13 protein-coding genes, ATG (*atp8*, *cytb*, *cox1-3*, *nad1*, *nad4L* and *nad5*), ATT (*atp6* and *nad6*), GTG (*nad2* and *nad4*) and ATA (*nad3*) are used as starting codons; TAA (*atp8*, *atp6*, *nad1*, *nad 3-6* and *nad4L*), TA (*cox3*) and T (*cytb* and *cox1-2*) serve as terminal codons. The mitogenome shows a classic crab-type gene arrangement regulation (Yang et al. 2010). The total AT content of the mitogenome is 69.8%.

A phylogenetic tree was constructed based on 12 concatenated protein-coding genes (except *nd6*) from 12 crab species , using maximum likelihood (ML) method. *Harpiosquilla harpax* was used as an outgroup for tree rooting (Figure 1). It was demonstrated that Grapsidae crabs formed a solid monophyletic group (99% bootstrap replicates). This mitochondrial genome provides genetic markers for studies on phylogeny of the grapsid crabs, which will be a part of mitochondrial genome library for evolutionary and systematic studies.

**Figure 1.**
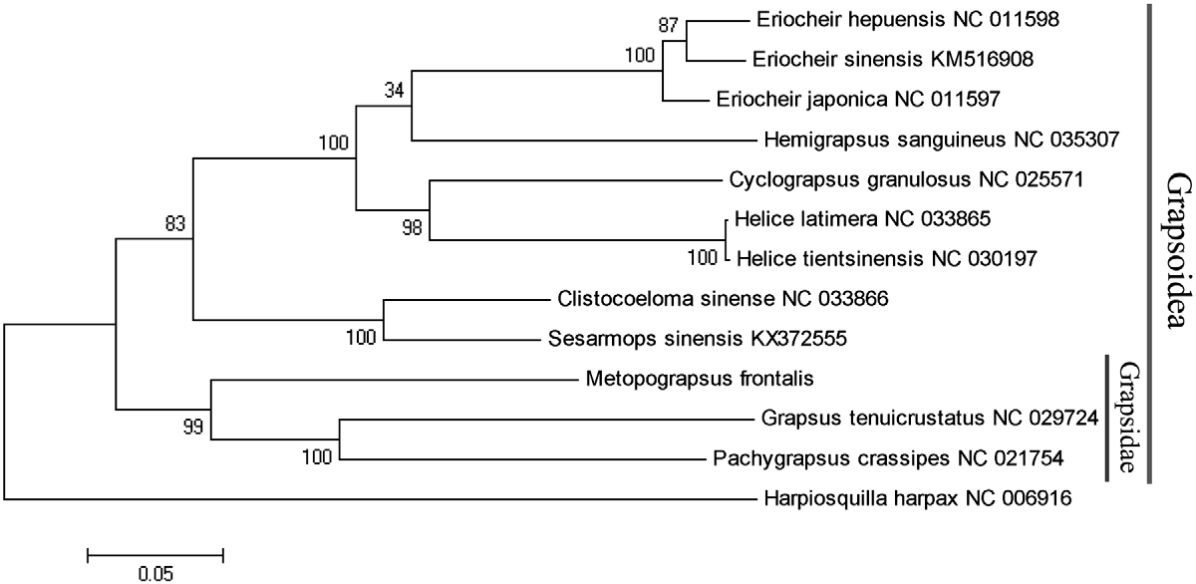
Phylogenetic tree of *M. frontalis* and related species based on maximum likelihood (ML) method. *Harpiosquilla harpax* was used as an outgroup.
